# Alteration of the N^6^-methyladenosine methylation landscape in a mouse model of polycystic ovary syndrome

**DOI:** 10.1186/s13048-023-01246-7

**Published:** 2023-08-08

**Authors:** Lingxiao Zou, Waixing Li, Dabao Xu, Shujuan Zhu, Bin Jiang

**Affiliations:** Department of Obstetrics and Gynaecology, The Third Xiangya Hospital of Central South University, 138 Tongzipo Road, Changsha, China

**Keywords:** N^6^-methyladenosine, mRNA, Polycystic ovary syndrome, Epitranscriptomic microarray, PI3K/AKT pathway

## Abstract

**Objective:**

To explore the N^6^-methyladenosine (m^6^A) methylation abnormality of mRNAs and its potential roles in the mouse model of polycystic ovary syndrome (PCOS).

**Methods:**

The mouse model of PCOS were induced by injecting dehydroepiandrosterone (DHEA), and confirmed by observing the morphological structures of ovarian follicles. Subsequently, m^6^A-tagged mRNAs were identified via m^6^A epitranscriptomic microarray and its potential functional pathways were predicted in KEGG database. The expression and modification levels of key mRNAs in the most enriched pathway were evaluated and compared using western blot and methylated RNA immunoprecipitation-quantitative PCR (MeRIP-qPCR).

**Results:**

Compared with the control group, 415 hypermethylated and downregulated mRNAs, 8 hypomethylated and upregulated mRNAs, and 14 hypermethylated and upregulated mRNAs were identified in the PCOS group (Fold change ≥ 1.5). Those mRNAs were mainly involved in insulin signaling pathway, type II diabetes mellitus, Fc epsilon RI signaling pathway, inositol phosphate metabolism, and GnRH secretion. In insulin signaling pathway, the expression levels of phosphorylated protein kinase B (p-AKT) were decreased, whereas that of upstream phosphorylated phosphatidylinositol 3-kinase (p-PI3K) were increased in PCOS group. Moreover, skeletal muscle and kidney-enriched inositol polyphosphate 5-phosphatease (SKIP), one of PIP3 phosphatases, was verified to be overexpressed, and *Skip* mRNAs were hypermethylated in PCOS group.

**Conclusion:**

The altered m^6^A modification of mRNAs might play a critical role in PCOS process. The PI3K/AKT pathway is inhibited in the mouse model of PCOS. Whether it is caused by the m^6^A modification of *Skip* mRNAs is worthy of further exploration.

**Supplementary Information:**

The online version contains supplementary material available at 10.1186/s13048-023-01246-7.

## Introduction

Polycystic ovary syndrome (PCOS) is a common endocrine and metabolic dysfunction condition in the women of reproductive age, resulting in irregular menstruation, hyperandrogenism, infertility, and insulin resistance(IR) [1]. Patients with PCOS are at higher risk of diabetes [2], cardiovascular disease [3], and endometrial cance [4]. There is no specific therapy for PCOS, only symptomatic treatment such as lifestyle management, taking combination oral contraceptives to regulate menstrual cycles and ameliorate hyperandrogenism, using insulin sensitizers to alleviate IR, taking clomiphene or letrozole to promote ovulation in the case of anovulatory infertility [5–7].

The etiology of PCOS is not yet entirely understood. It is widely assumed that PCOS is caused by a combination of environmental and genetic factors. PCOS is associated with hypothalamic-pituitary-ovarian axis (HPOA) neuroendocrine abnormalities, hyperandrogenemia, IR, chronic inflammation, and circadian rhythm disorder [5, 8]. At the same time, PCOS appears to be a highly genetic and polygenic disease. Despite the fact that candidate gene association studies and genome-wide association studies have identified several susceptibility genes and single-nucleotide polymorphism loci significantly associated with PCOS, such as follicle stimulating hormone receptor gene (*FSHR*, rs2268361, rs2349415), insulin receptor (*INSR*, rs2059807), it is still difficult to explain the complexity of PCOS etiology and clinical manifestations [9–11]. Recently, epigenetic factors have received a lot of attention in the pathogenesis of PCOS. As far as we know,women with PCOS have different epigenetic regulation, including DNA methylation, histone acetylation, and changes in non coding RNA content [12, 13]. The changes of DNA methylation in peripheral and umbilical cord blood, ovary and adipose tissue of PCOS patients indicate that this epigenetic modification is related to the pathogenesis of the disease [14]. Perhaps, these defects in DNA methylation promote the disorder of genes involved in inflammation, hormone synthesis and signal transduction, as well as glucose and lipid metabolism [15–19]. And the research on the role of DNA methylation in the pathogenesis of PCOS has just begun.

N^6^-methyladenosine (m^6^A) is the most prevalent internal modification of mRNAs, which is reversible and dynamically regulated by methyltransferase complexes (writers) and demethylases (erasers), and recognized by m^6^A binding proteins (readers) [20]. m^6^A modification regulates post-transcriptional expression of m^6^A-tagged genes by participating in RNA metabolism such as pre-mRNA splicing, mRNA translation, nuclear export, mRNA decay, and non-coding RNA biogenesis [21]. Recently, studies have revealed the role of m^6^A modifications and their protein machines in oogenesis and in female reproductive tumors, as well as in other female reproductive diseases [22]. It has been demonstrated that the m^6^A modification level was increased, and several m^6^A modulators were dysfunction in PCOS patients [23]. In oogenesis, lack of YTHDF2 leads to failure of m6A modified mRNA degradation, which affecting the oocyte quality [24, 25]. The m^6^A proteins are also involved in ovulation, and the m^6^A writers play an important role in oogenesis, but whether they can be used as therapeutic targets for abnormal ovulation remains to be further investigated. Nevertheless, little attention has been paid to the molecular mechanisms of m^6^A modification in PCOS.

In this study, we aim to investigate the altered m^6^A modification landscape of mRNAs in the ovaries of PCOS mice using the epitranscriptomic microarray, and to preliminarily explore the potential signal pathways involved in the PCOS process though KEGG analysis.

## Materials and methods

The study was approved by the Institutional Review Board (No.2021-S087), and animal experiments were in accordance with the Guide for the Care and Use of Laboratory Animal by International Committees of The Third Xiangya Hospital of Central South University.

### Establishment of a mouse model of PCOS

Female C57BL/6J mice aged 7 weeks were acquired from SJA Laboratory Animal Co. Ltd (Hunan China), and adaptively fed for 1 week on 12 h light/ 12 h dark cycle at room temperature (24 ± 3 °C) with a humidity of 45 ± 2%. During the feeding period, vaginal smears were performed daily at 8:00 a.m. to observe the estrous cycle of mice. Ten mice with normal estrous cycle were selected and randomly divided in two groups: the control and PCOS group. In the PCOS group, 6 mg/(100 g·d) dehydroepiandrosterone (DHEA) and 0.2 ml injectable soybean oil were injected subcutaneously into mice daily for 20 consecutive days. Similarly, mice in the control group were injected with 0.2 ml injectable soybean oil daily for 20 days.

Subsequently, five mice with continuous keratosis of vaginal epithelial cells in PCOS group and five mice in control group were randomly sacrificed on the first day. The two ovaries randomly selected mice from each group were embedded in paraffin and sliced for hematoxylin and eosin (HE) staining to observe the morphological changes and confirm the induction of PCOS. And the three remaining mice in each group, the left ovaries were preserved at -80 °C for following N^6^-methyladenosine detection, the right ovaries were preserved at -80 °C for following western blot.

### RNA extraction and quality control

To isolate total RNA, ovary tissues were cut into small pieces and homogenized in TRIzol reagent before being quantified using a NanoDrop ND-1000 (Thermo Fisher Scientific, Waltham, MA, USA). Supplementary Table S1 presents the quantification and quality of RNA.

### m^6^A immunoprecipitation

Total RNA (1-3ug) mixed with m^6^A spike-in control was immunoprecipitated with anti-m^6^A rabbit polyclonal antibody (Synaptic Systems, Göttingen, Germany) at 4 °C for 2 h. Dynabeads™ M-280 Sheep Anti-Rabbit IgG suspension (20 μL per sample) (Ivitrogen) was blocked with 0.5% bovine serum albumin (BSA) at 4 °C for 2 h, washed three times with IP buffer (300 μL) for 5 min, and resuspended in the prepared RNA-antibody mixture. The RNA was bound to the m^6^A-antibody beads for 2 h at 4 °C, then the beads were washed with IP buffer (500 μL, three times), followed by Wash buffer (500 μL, twice). In this case, the adsorbed RNA was eluted with Elution buffer (200 μL) at 50 °C for 1 h. The immunoprecipitated (IP) RNA and supernatant (Sup) RNA were extracted by acid phenol–chloroform and ethanol precipitation.

### Labeling and hybridization

The IP RNAs and Sup RNAs were mixed with an equal amount of calibration spike-in control RNA, amplified separately and labeled with Cy3 (for Sup) and Cy5 (for IP) using Arraystar Super RNA Labeling Kit (Arraystar). The synthesized cRNAs was purified by the RNeasy Mini Kit (QIAGEN), and the concentration and specific activity of cRNAs were detected by the NanoDrop ND-1000 (Thermo Fisher Scientific) (Supplementary Table S2). 2.5 μg of Cy3 and Cy5-labeled cRNAs were mixed, added with 5 μL 10 × Blocking Agent and 1 μL of 25 × Fragmentation Buffer. It was heated to 60℃ for 30 min, and then mixed with 25 μL 2 × Hybridization buffer. 50 μl of hybridization solution was injected into the gasket slide and assembled on the m^6^A-mRNA epitranscriptome microarray slide. The slides were incubated at 65 °C for 17 h in an Agilent Hybridization Oven (Agilent, CA, USA). The hybridized arrays were washed, fixed, and scanned with an Agilent scanner G2505C (Agilent).

### Epitranscriptomic microarray data analysis

To analyze acquired array images, Agilent Feature Extraction software (version 11.0.1.1) was used. The raw IP and Sup intensities were normalized to the log2-scaled Spike-in RNA intensity average. Following Spike-in normalization, probe signals with Present (P) or Marginal (M) QC flags were retained in at least three of six samples for further m^6^A methylation, quantity, and expression level analyses. The m^6^A methylation level was calculated for the percentage of modification based on the IP and Sup normalized intensities, and the m^6^A quantity was calculated for the m^6^A methylation amount. The expression level was calculated by adding the IP and Sup normalized intensities, and an additional quantile normalization method from the limma package was used to normalize the RNA expression level between arrays before flagging probes.

### Methylated RNA immunoprecipitation-quantitative PCR (MeRIP-qPCR)

The m^6^A epitranscriptomic microarray data was validated using IP (*n* = 3, each group). MeRIP-qPCR was then used to quantify the RNA enrichment via 2^−ΔΔct^ analysis. Supplementary Table S3 describes the primer used. Furthermore, the mRNA m^6^A sites were predicted using the sequence-based RNA adenosine methylation site predictor (SRAMP) program (https://www.cuilab.cn/sramp) [26].

### Western blot

The protein concentration of right ovary tissue was determined using the Bradford method after protein extraction (M&C Gene Technology Ltd.). SDS–polyacrylamide gel electrophoresis (SDS-PAGE) was used to separate the protein samples, which were then transferred to a polyvinylidene difluoride membrane. Antibodies of interest were used to probe the membranes. The antibodies used were as follows: phosphatidylinositol 3-kinase (PI3K) (1:1000; ABclonal), phosphorylated PI3K (p-PI3K) (1:1000; ABclonal), protein kinase B (AKT) (1:1000; Proteintech), phosphorylated AKT (ser473) (p-AKT) (1:1000; Proteintech), skeletal muscle and kidney-enriched inositol polyphosphate 5-phosphatase (SKIP) (1:1000; Proteintech), and β-actin (1:1000; Abcam).

### Statistical analysis

Filtering with the fold change ≥ 1.5 and statistical significance thresholds (*P* < 0.05) revealed differentially m^6^A-methylated or differentially expressed RNAs between two comparison groups. Hierarchical clustering was carried out using the R software (version 4.02). To perform GO analysis, the topGO package in the R environment for statistical computing and graphics was used, and the Fisher's exact test was used to perform pathway analysis. The western blot data were presented as mean ± SD and compared between groups using the Student's t test or the Mann–Whitney U test. *P* < 0.05 was regarded as significant. SPSS version 21.0 was used for all statistical analysis (SPSS 21.0, Inc.,Chicago, IL, USA).

## Results

### Mouse models of PCOS

**Fig. 1:**
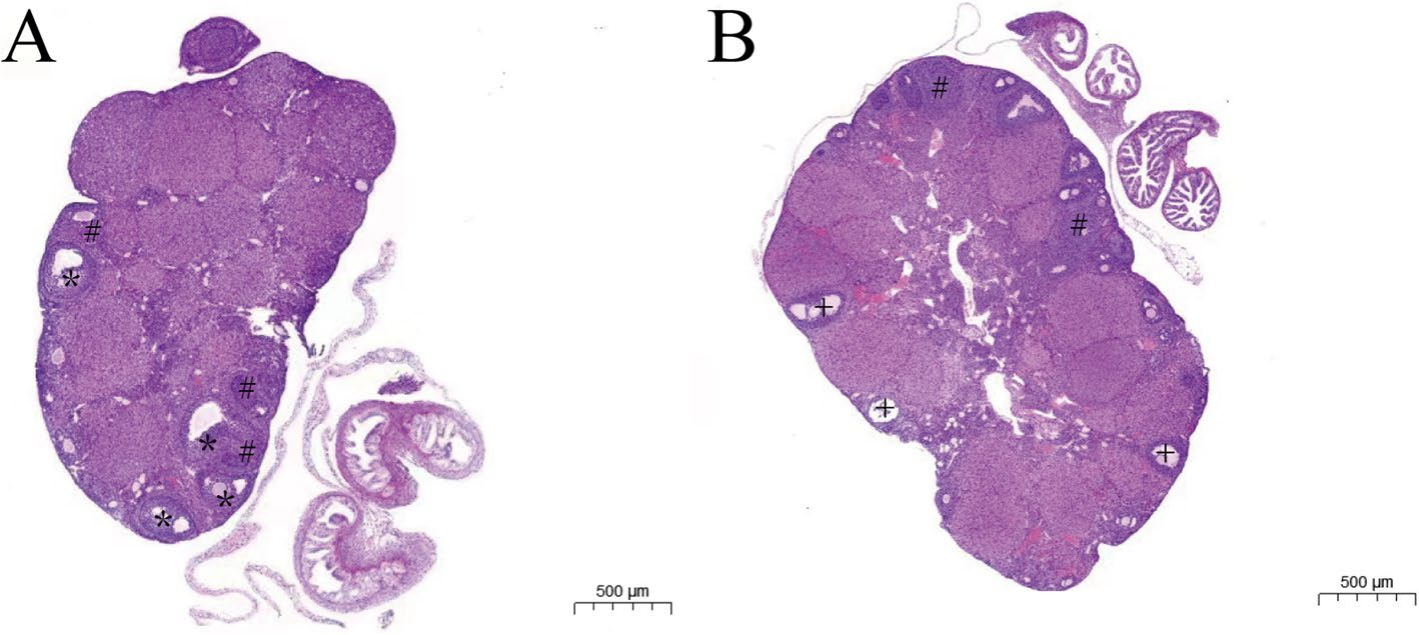
The hematoxylin and eosin (HE) staining of ovaries of mouse model in two groups.** A** the control group; **B** the PCOS group. *: developing follicles; #: corpus luteum; + : cystic follicles

In the control group, different stages of follicular development were seen in ovaries, and the ovarian morphology was normal without large cysts (Fig. 1A); While the ovaries were swollen with multiple cystic follicles in the PCOS group, and there were cyst-like expanded follicles on the surface of ovaries (Fig. 1B). These indicated that the mouse model of PCOS was successfully established.

### Identification of differentially expressed or methylated genes

**Fig. 2:**
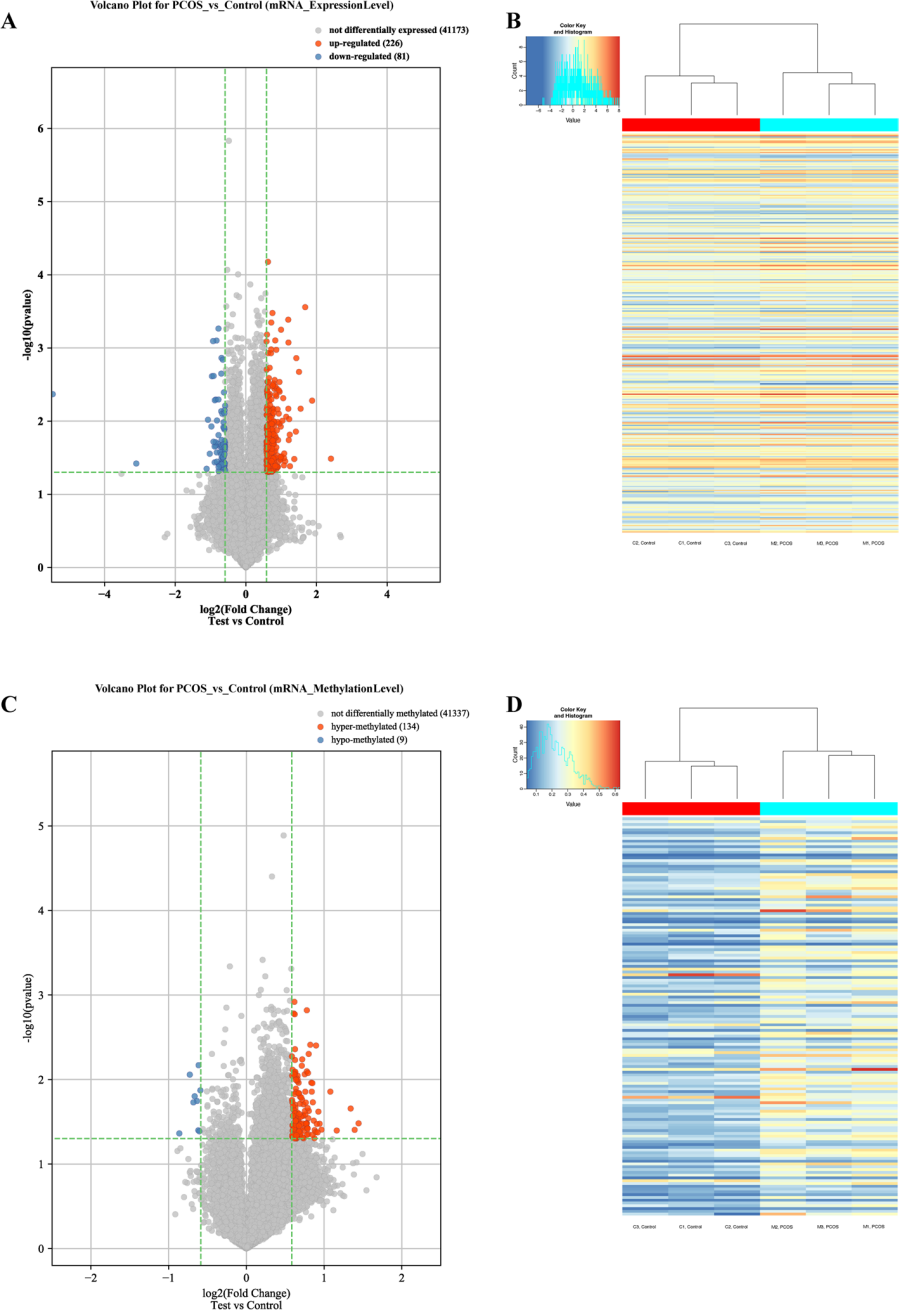
The volcano plot and heatmap plot of differentially expressed or methylated mRNAs. **A** volcano plot of differentially expressed mRNAs. **B** heatmap plot of differentially expressed mRNAs. **C** volcano plot of differentially methylated mRNAs. **D** heatmap plot of differentially methylated mRNAs

The microarray results showed that 307 mRNAs were significantly differentially expressed (Fold change ≥ 1.5, *P* value < 0.05) between the PCOS and control groups. Among them, 226 and 81 mRNAs were upregulated and downregulated in the PCOS group, respectively (Fig. 2A and B). Ten most significantly differentially regulated mRNAs are listed respectively in Table 1.

**Table 1:** Significantly differentially expressed mRNAs

ProbeName	Regulation	Gene Symbol	Fold change	RNA length	*P* value
ASMM10EP3A100132658	up	*Gpr82*	5.3153184	2497	0.032583289
ASMM10EP3A100185262	up	*Myf5*	3.6723207	2083	0.005246494
ASMM10EP3A100023189	up	*Rp24-399b3.5*	3.2057885	2578	0.00027672
ASMM10EP3A100077632	up	*Adamts5*	2.9303902	8091	0.006757898
ASMM10EP3A100095162	up	*Bmp2*	2.8393637	3555	0.002122044
ASMM10EP3A101254336	down	*Myom1*	0.6666035	4069	0.046797259
ASMM10EP3A122804969	down	*Rad1*	0.6658879	1308	0.006171832
ASMM10EP3A115300704	down	*Ap2a2*	0.6657283	590	0.020065485
ASMM10EP3A128360468	down	*Tnfaip1*	0.6651064	3720	0.022267455
ASMM10EP3A104603562	down	*Cyp11a1*	0.6643443	539	0.024616172

Comparing with the m^6^A levels in the control groups, 143 mRNAs had significantly differential modification levels (Fold change ≥ 1.5, *P* value < 0.05) in the PCOS group. Surprisingly, of these 143 mRNAs, 134 mRNAs were found to have higher m^6^A methylation levels and only 9 mRNAs had lower levels of m^6^A methylation (Fig. 2C and D). Ten most significantly differentially m^6^A-methylated mRNAs are listed in Table 2.

**Table 2:** Significantly differentially methylated mRNAs

Gene Symbol	Regulation	Fold change	*P* value	FDR
*Cracr2a*	hyper	2.7203737	0.032991375	0.9997448
*Zranb3*	hyper	2.6272047	0.039447088	0.9997448
*Grik2*	hyper	2.5353444	0.022043029	0.9997448
*Rnf39*	hyper	2.2392177	0.040153637	0.9997448
*Pcnxl2*	hyper	2.1146009	0.013918031	0.9997448
*Etfrf1*	hypo	0.6634367	0.0134237	0.9997448
*Igdcc4*	hypo	0.6578386	0.0410282	0.9997448
*Tmeff2*	hypo	0.6529331	0.0067753	0.9997448
*Ac138587.1*	hypo	0.6514125	0.0399025	0.9997448
*Meis3*	hypo	0.6435131	0.0179554	0.9997448

Moreover, we found several “writers” and “readers” of m^6^A modification were up-regulated in PCOS group, including YTH N6-methyladenosine RNA binding protein 3 (*Ythdf3*) (Foldchange = 1.35, *P* < 0.05), heterogeneous nuclear ribonucleoprotein A2/B1 (*Hnrnpa2b1*) (Foldchange = 1.615713434, *P* < 0.05).

### Analysis of differentially expressed with differentially methylated genes

**Fig. 3:**
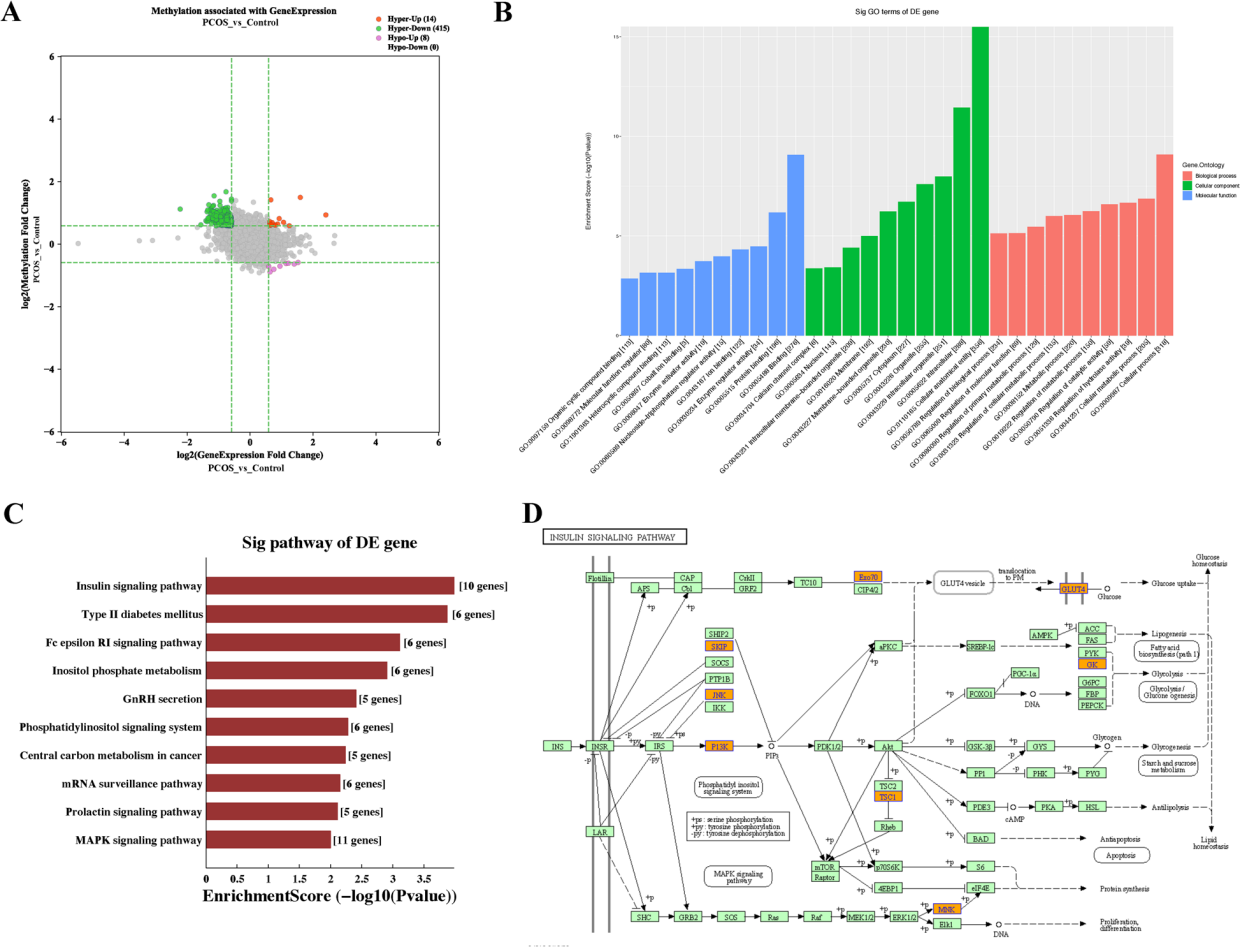
Analysis of differentially expressed with differentially methylated genes. **A**. scatter plot; **B**. the top ten enriched items obtained from GO analysis; **C**. the first ten enriched pathways identified in KEGG analysis. **D**. the most enriched pathway-insulin signaling pathway. mRNAs with different expression and m^6^A modification levels were marked in orange. The diagram is based on the insulin signaling pathway in the Kyoto encyclopedia of genes and genomes pathway database

When we integrated the mRNAs methylation and expression data, 8 hypomethylated and upregulated mRNAs, 415 hypermethylated and downregulated mRNAs, 14 hypermethylated and upregulated mRNAs, and 0 hypomethylated and downregulated mRNAs (Fold change ≥ 1.5) were identified (Fig. 3A). Then, the functions of these 437 mRNAs were analyzed through GO and KEGG pathway analyses. Among the enriched GO terms, “cellular process” in biological process (BP), “cellular anatomical entity” in cellular components (CC), “binding” in molecular function (MF) earned the highest enrichment score (Fig. 3B). As for the KEGG analysis, mRNAs were predicted to participated in 24 pathways and the most enriched ten pathways are shown in Fig. 3C. Insulin signaling pathway, the most enriched pathway, involved ten differentially expressed mRNAs with differentially methylation (Fig. 3D).

**Fig. 4:**
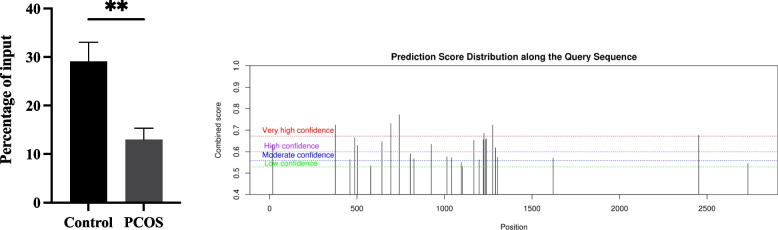
The comparison of m^6^ A modification levels and the predicted m^6^ A site of *Skip* mRNAThe left image shows that the m^6^ A modification levels were higher in the PCOS group than that in the control group (*P* < 0.01); The right image shows that the predicted m.^6^ A sites by SRAMP program (https://www.cuilab.cn/sramp)

Among these, the higher m^6^A methylation levels of the skeletal muscle and kidney-enriched inositol polyphosphate 5-phosphatase (*Skip*) mRNA were verified via MeRIP-PCR (*P* < 0.01) (Fig. 4). Moreover, analysis of *Skip* mRNA with SRAMP program predicted six potential m^6^A sites with very high confidence, including five sites on the coding sequence and one sites on the 3’untranslated region (UTR) (Fig. 4, Supplementary Table 4).

### Suppressed phosphatidylinositol 3‑kinase (PI3K)/protein kinase B (AKT) signaling pathway

**Fig. 5:**
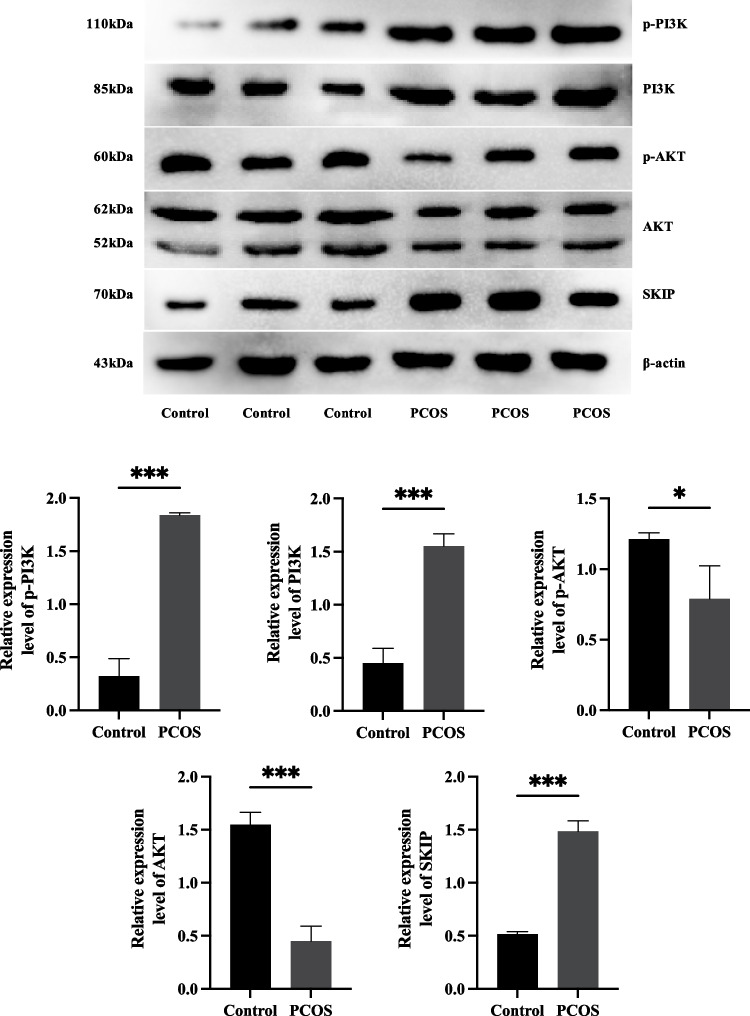
Suppressed PI3K/AKT signaling pathway and elevated SKIP expression. The top image shows the representative image of western blotting in control and PCOS group; The remaining images shows the comparison of PI3K, p-PI3K, AKT, p-AKT, and SKIP expression levels between two groups. Data were shown as the mean ± SD. All experiment was conducted in triple. *P < 0 .05, **P < 0.01 and ***P < 0.001

We further explored the activity levels of PI3K/AKT signaling pathway by western blotting, which is one part of insulin signaling pathway. It demonstrated that the expression of AKT and p-AKT (ser473) were significantly decreased (*P* < 0.001), whereas the expression of PI3K and p-PI3K were significantly increased (*P* < 0.05) in PCOS group than that in control group (Fig. 5). Notably, the SKIP, a phosphatidylinositol 3,4,5-trisphpsphate (PIP3) phosphatase, was identified to be overexpressed in PCOS group (*P* < 0.001) (Fig. 5).

## Discussion

Polycystic ovary syndrome (PCOS) has numerous adverse effects on women. However, the pathogenesis of PCOS is currently unclear, and there is no specific therapy for PCOS. Given the continuously increasing incidence of PCOS in recent years, it is important to explore the mechanisms of PCOS development and to develop effective treatments for patients with PCOS. PCOS is commonly associated with aberrant DNA methylation, and several genes are epigenetically dysregulated, and are associated with the pathological consequences of PCOS and metabolic comorbidities [27]. However, the methylation status of specific genes and the extent to which genes are dysregulated in terms of methylation patterns are unknown.Due to the reversibility of epigenetic modifications, "druable" regions can be screened to target or correct abnormalities in gene expression, so PCOS methylation promises the development of novel chromatin methylation therapies targeting PCOS [28].Herein, we used epitranscriptomic microarray for the first time to investigate the altered m^6^A modification of mRNAs and preliminarily explore the potential molecular mechanisms of m^6^A modification in the mouse model of PCOS induced by hyperandrogenism, and to screen new molecular targets and develop effective targets for treating PCOS or inhibiting its progression.

According to our microarray results, the m^6^A modification levels of mRNAs were increased, and the m^6^A ‘readers’ (*Ythdf3*, *Hnrnpa2b1*) are overexpressed in PCOS mice ovaries. A similar methylation trend was confirmed using MeRIP sequencing (MeRIP-seq) in luteinized granulosa cells (GCs) of PCOS patients [23]. In contrast to MeRIP-seq, epitranscriptomic microarray can determined the percentage of modified and unmodified RNA of each transcript [29]. As a result, 437 RNAs with differentially expressed and differentially methylated levels were identified. Further bioinformatics analysis revealed that the m^6^A modification may mainly participate in the insulin signaling pathway and type II diabetes mellitus pathway, which were closely related to IR and secondary hyperinsulinemia [30]. The PI3K/AKT pathway and the mitogen activated protein kinase (MAPK) pathway were two major insulin-related signal transduction pathway, regulating the glucose metabolism, cell proliferation and differentiation, respectively [30, 31]. In mouse cumulus-oocyte complexes, activated PI3K/AKT signaling can increase glucose uptake by mediating the translocation of GLUT4 to the GCs membrane, which provides energy substrate for follicular development [32]. The dysfunction of PI3K/AKT signaling is not only linked to IR, inflammation, and oxidative stress, but it also inhibits proliferation and promotes apoptosis, all of which may contribute to PCOS [33, 34]. Similarly, the inhibition of PI3K/AKT pathway in PCOS mice was confirmed in our study by assessing p-AKT expression levels.

Notably, the activity of upstream and downstream factors in the PI3K/AKT pathway were opposite. In contrast to p-AKT, p-PI3K expression levels were elevated in the PCOS group, which may be related to inositol phosphate metabolism pathway, another enriched pathway. SKIP, a PIP3 5-phosphatase, was localized at endoplasmic reticulum under resting conditions. Insulin stimulation induced its translocation to the plasma membrane, and binding with activated p21-activated protein kinase 1 (PAK1), thereby activating SKIP’s PIP3 phosphatase activity. The rapid and efficient hydrolysis of PIP3, resulting in decreased AKT2 phosphorylation, inhibits membrane ruffle and GLUT4 translocation, thus negatively regulates insulin signaling in skeletal muscle [35, 36]. As expected, SKIP protein was confirmed to be significantly overexpressed in PCOS mice in this study. Furthermore, due to the non-statistically significant difference in mRNA expression levels between two groups, SKIP overexpression appears to be linked to higher m^6^A modification in PCOS mice. The m^6^A sites in humans and mice are highly conserved, and mainly enriched in the 3’untranslated region (UTR) and around stop codons [37]. However, Shen Zhang et al. discovered increased m^6^A peaks in the coding sequence and transcription start regions, but less prominent enrichment near stop codons in PCOS patients' GCs compared to controls [23]. Similarly, the m^6^A sites of *Skip* mRNA was also mainly predicted in the coding sequence by SRAMP program in this study [26]. In addition, previous research revealed that the METTL3-induced m^6^A modification in the coding sequence may alleviate ribosome stalling and thus improve mRNA translation in acute myeloid leukemia [38]. And in Arabidopsis thaliana, increased mRNA expression levels were associated with a modification change around the start codon [39]. Therefore, the altered m^6^A modification of *Skip* mRNA may explain its translation upregulation. Therefore, it is possible that the higher m^6^A modification of *Skip* mRNA result in the overexpression of SKIP protein, then reversed the activation of p-PI3K on the downstream AKT.

In addition, Fc epsilon RI signaling pathway and GnRH secretion pathway, another two enriched pathways, may indicated that the m^6^A modification also participant in the etiological mechanism of chronic inflammation and neuroendocrine in PCOS. Increasing number of studies demonstrated that low-grade chronic inflammation can induce IR, obesity, and hyperandrogenemia through related pathways, leading to ovulation disorders in PCOS [40–42]. The HPOA plays an important role in the regulation of female reproductive endocrine as an integrated and coordinated neuroendocrine system. Hypothalamic gonadotropin-releasing hormone (GnRH) neurons secrete GnRH, which regulates the secretion of gonadotropins FSH and LH, then regulate the secretion of sex hormones and reproductive function [43]. The GnRH neuronal firing activity was identified to be increased in PCOS mice induced by prenatal androgenization compared to normal mice, which may be closely linked to PCOS development [44, 45].

Inevitably, there were several limitations of this study. First, several acknowledged inhibiting factors for PI3K/AKT pathway were not investigated, such as Phosphatase and tensin homolog (PTEN) and c-Jun N-terminal kinase (JNK). Then, we did not further validate our hypothesis that the hypermethylation of SKIP mRNAs enhances its expression, then inhibits the PI3K/AKT signaling pathway.

In conclusion, our study demonstrates that the altered m^6^A modification of mRNAs might play a critical role in PCOS process. And we emphasized the changes in the activity of upstream and downstream factors in the PI3K/AKT signaling pathway. Moreover, the role of m^6^A modification of *Skip* mRNA in the pathogenesis of PCOS warrants further studies.

### Supplementary Information

**Additional file 1:** **Supplementary Table S1. **the quantification and quality of RNA in ovary tissue of a mouse model of PCOS*. **Supplementary Table S2. **The specific activity (pmol dyes per μg cRNA) of the labeled RNA. **Supplementary Table S3. **Primers used in MeRIP-qPCR. **Supplementary Table S4.** Predicted m^6^A sites in *Skip* mRNA by SRAMP program.

**Additional file 2:** **Supplementary Figure S1. **Characteristics of vaginal smears during various stages of estrus cycle  A. Proestrous, which was characterized by the presence of mostly nucleated and some cornified epithelial cells; B. Estrous, which was characterized by the presence of mostly cornified epithelial cells; C. Metestrus, which was characterized by the presence of cornified epithelial cells and leukocytes; D. Diestrus, which was characterized by the presence of primarily leukocytes.

## Data Availability

The data and material presented in this manuscript is available from the corresponding author on reasonable request.
