# Correction: Alteration of the N^6^‑methyladenosine methylation landscape in a mouse model of polycystic ovary syndrome

**DOI:** 10.1186/s13048-024-01447-8

**Published:** 2024-06-08

**Authors:** Lingxiao Zou, Waixing Li, Dabao Xu, Shujuan Zhu, Bin Jiang

**Affiliations:** Department of Obstetrics and Gynaecology, The Third Xiangya Hospital of Central South University, 138 Tongzipo Road, Changsha, China

**Correction** **:** **J Ovarian Res 16, 157 (2023)**

10.1186/s13048-023-01246-7

Following publication of the original article [1], the authors reported that Fig. 5 image in the published article showed garbled characters. See below correct Fig. 5.

Incorrect:


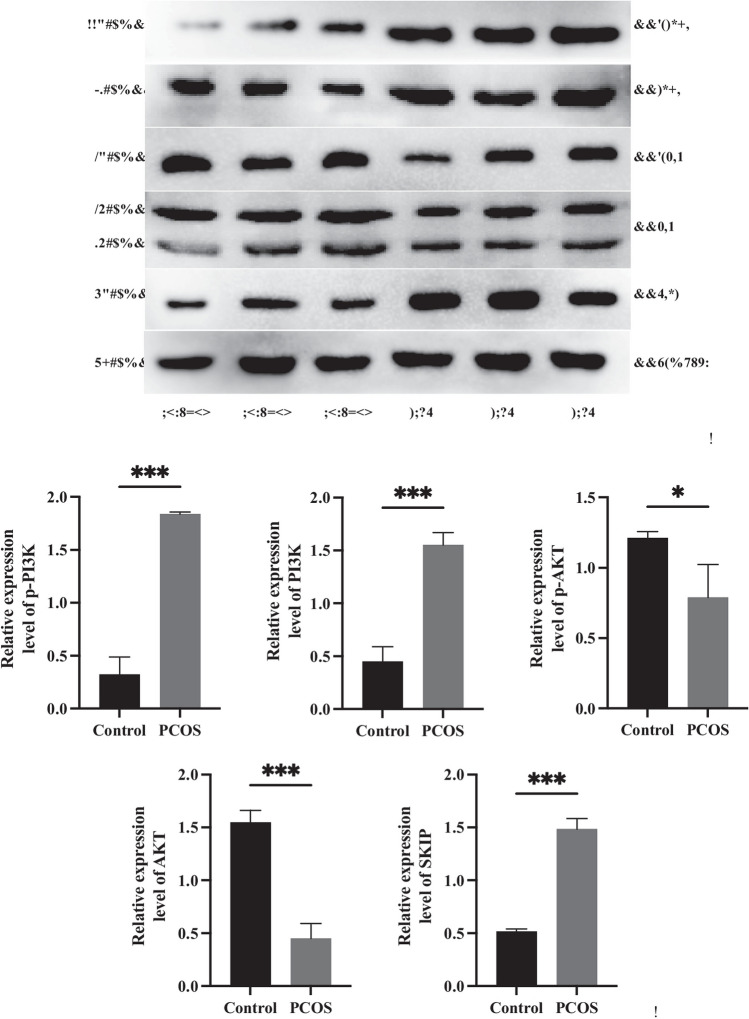


Correct:


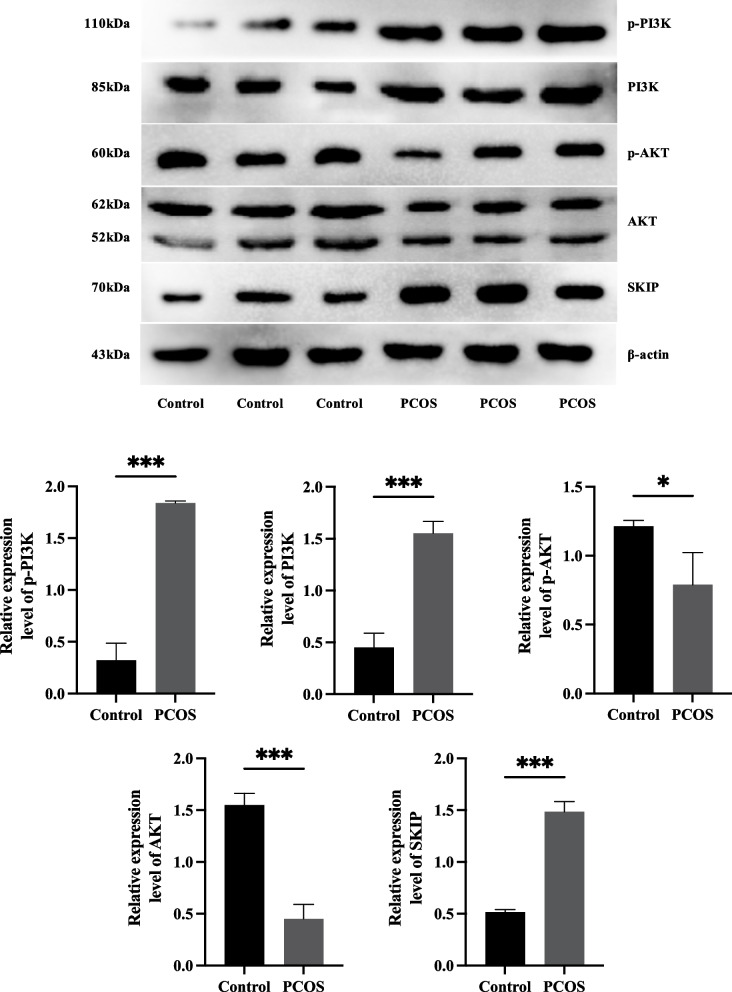


The original article has been corrected.
