# Supplementary figures and images for: Alteration of the N6-methyladenosine methylation landscape in a mouse model of polycystic ovary syndrome
Source: J Ovarian Res. 2023 Aug 8;16:157. doi: 10.1186/s13048-023-01246-7 (PMC10408202; doi:10.1186/s13048-023-01246-7)

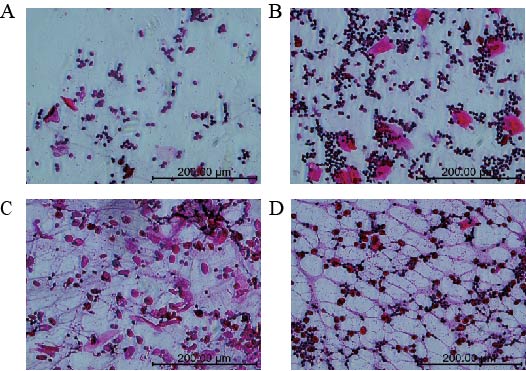

Supplement: Supplementary file 1 — Additional file 1: Supplementary Table S1. the quantification and quality of RNA in ovary tissue of a mouse model of PCOS*. Supplementary Table S2. The specific activity (pmol dyes per μg cRNA) of the labeled RNA. Supplementary Table S3. Primers used in MeRIP-qPCR. Supplementary Table S4. Predicted m6A sites in Skip mRNA by SRAMP program. [file 13048_2023_1246_MOESM1_ESM.jpg]
