# Supplementary material for: Alteration of the N6-methyladenosine methylation landscape in a mouse model of polycystic ovary syndrome
Source: J Ovarian Res. 2023 Aug 8;16:157. doi: 10.1186/s13048-023-01246-7 (PMC10408202; doi:10.1186/s13048-023-01246-7)
Supplement: Supplementary file 2 — Additional file 2: Supplementary Figure S1. Characteristics of vaginal smears during various stages of estrus cycle A. Proestrous, which was characterized by the presence of mostly nucleated and some cornified epithelial cells; B. Estrous, which was characterized by the presence of mostly cornified epithelial cells; C. Metestrus, which was characterized by the presence of cornified epithelial cells and leukocytes; D. Diestrus, which was characterized by the presence of primarily leukocytes. [file 13048_2023_1246_MOESM2_ESM.pdf]

**Supplementary Table S1** the quantification and quality of RNA in ovary tissue of a mouse model of PCOS*

| Sample ID | OD260/280 Ratio | OD260/230 Ratio | Conc. (ng/µl) | Volume (µl) | Quantity (ng) | QC Purity Pass or Fail |
|---|---|---|---|---|---|---|
| Control 1 | 1.99 | 2.01 | 551.83 | 20 | 11036.60 | Pass |
| Control 2 | 1.99 | 2.37 | 676.63 | 40 | 27065.20 | Pass |
| Control 3 | 1.98 | 2.45 | 712.99 | 40 | 28519.60 | Pass |
| PCOS 1 | 1.97 | 2.41 | 795.43 | 20 | 15908.60 | Pass |
| PCOS 2 | 1.98 | 2.36 | 650.87 | 40 | 26034.80 | Pass |
| PCOS 3 | 1.96 | 2.48 | 673.65 | 40 | 26946.00 | Pass |

*For spectrophotometer, the O.D. A260 /A280 ratio should be close to 2.0 for pure RNA (ratio between 1.8 and 2.1 are acceptable). The O.D. A260/A230 ratio should be more than 1.8.

**Supplementary Table S2** The specific activity (pmol dyes per μg cRNA) of the labeled RNA

| Sample ID | Dye Name | Dye pmol/μl | cRNA Concentration (μg/μl) | Specific Activity*(pmol Dye/μg cRNA) | Volume (μl) | Total Amount (μg) |
|---|---|---|---|---|---|---|
| PCOS 1 Sup | Cy3 | 11.93 | 0.78 | 15.22 | 10.00 | 7.84 |
| PCOS 2 Sup | Cy3 | 10.15 | 0.69 | 14.65 | 10.00 | 6.93 |
| PCOS 3 Sup | Cy3 | 11.78 | 0.74 | 15.99 | 10.00 | 7.37 |
| Control 1 Sup | Cy3 | 13.34 | 0.86 | 15.58 | 10.00 | 8.56 |
| Control 2 Sup | Cy3 | 12.13 | 0.79 | 15.28 | 10.00 | 7.94 |
| Control 3 Sup | Cy3 | 11.53 | 0.75 | 15.44 | 10.00 | 7.47 |
| PCOS 1 IP | Cy5 | 4.84 | 0.41 | 11.79 | 10.00 | 4.11 |
| PCOS 2 IP | Cy5 | 4.23 | 0.38 | 11.00 | 10.00 | 3.84 |
| PCOS 3 IP | Cy5 | 2.88 | 0.29 | 9.85 | 10.00 | 2.92 |
| Control 1 IP | Cy5 | 2.83 | 0.27 | 10.48 | 10.00 | 2.70 |
| Control 2 IP | Cy5 | 4.02 | 0.38 | 10.48 | 10.00 | 3.84 |
| Control 3 IP | Cy5 | 2.95 | 0.28 | 10.44 | 10.00 | 2.83 |

*For two-color, if the yield is <825 ng and the specific activity is <8.0 pmol Cy3 or Cy5 per μg cRNA do not proceed to the hybridization step. Repeat cRNA preparation.

**Supplementary Table S3** Primers used in MeRIP-qPCR

| Gene | Forward primer | Reverse primer | Product length (bp) |
|---|---|---|---|
| *Skip* | 5’-CATTGCTGACTGTGCCTTCG-3’ | 5’-ATAGCCCAGAGCCTTGTGCC-3’ | 121 |
